# The Impact of Human Immunodeficiency Virus (HIV) Co-Infection on the Economic Burden of Cutaneous Leishmaniasis (CL) in Brazil and Potential Value of New CL Drug Treatments

**DOI:** 10.4269/ajtmh.13-0309

**Published:** 2014-09-03

**Authors:** Stephanie D. Kruchten, Kristina M. Bacon, Bruce Y. Lee

**Affiliations:** Department of Epidemiology, Graduate School of Public Health, University of Pittsburgh, Pittsburgh, Pennsylvania; Public Health Computational and Operations Research (PHICOR), Johns Hopkins Bloomberg School of Public Health, Baltimore, Maryland

## Abstract

Convergence of geographic regions endemic for human immunodeficiency virus (HIV) and cutaneous leishmaniasis (CL) raise concerns that HIV co-infection may worsen CL burden, complicating already lengthy and costly CL treatments and highlighting a need for newer therapies. We constructed two Markov decision models to quantify impact of HIV on CL and help establish a target product profile for new CL treatments, accounting for co-infection. The HIV co-infection increased lifetime cost per CL case 11–371 times ($1,349–45,683) that of HIV-negative individuals ($123) and Brazil's CL burden from $1.6–16.0 million to $1.6–65.5 million. A new treatment could be a cost saving at ≤ $254 across several ranges (treatments seeking probabilities, side effect risks, cure rates) and continues to save costs up to $508 across treatment-seeking probabilities with a drug cure rate of ≥ 50%. The HIV co-infection can increase CL burden, suggesting more joint HIV and CL surveillance and control efforts are needed.

## Introduction

Substantial overlap of the geographic regions endemic for human immunodeficiency virus (HIV) and cutaneous leishmaniasis (CL) infections raise concerns that HIV co-infection may exacerbate the presentation and thus the economic burden of CL.^1^ The HIV-infection can increase susceptibility to CL infection and lead to atypical and more severe clinical manifestations of CL, such as more widespread, diffuse, and disfiguring lesions and even potentially affecting the bone marrow and internal organs such as the spleen or liver.^2^,^3^ Furthermore, many healthcare workers may lack the experience, training, or established procedures to properly diagnose and treat these unusual presentations and associated clinical course, leading to treatment delays and consequently worse outcomes (e.g., permanent disfigurement).^2^

Although our prior analysis quantified the impact of CL in addition to evaluating the potential cost-effectiveness of a CL vaccine, which could help guide the formulation of the vaccine's target product profile (TPP),^4^ this study did not account for CL/HIV co-infection, a relatively recent phenomenon. Currently, available CL treatments are already lengthy and costly; HIV co-infection further complicates these treatments and highlights the need for newer drug therapies.^2^ Therefore, we developed an economic model of CL with and without HIV co-infection to quantify the impact of HIV co-infection on CL infection and a second model to help establish a TPP for a new CL treatment, accounting for HIV co-infection.

## Methods

### Model overview.

Our models focused on Brazil, which accounts for 30% of all HIV cases, 40% of all CL cases in South America,^5^,^6^ and have the most comprehensive country-specific CL/HIV co-infection data found in the literature. Deforestation and urbanization of CL, once thought of as a strictly rural disease, along with dispersion of HIV beyond urban centers, has resulted in overlapping geographical distributions of CL and HIV^7^,^8^ and has led to the emergence of CL/HIV co-infection in the past two decades. Furthermore, mucocutaneous leishmaniasis (MCL), a more severe form of CL, is observed in up to 43% of *Leishmania*/HIV cases in Brazil, a rate much higher than in other regions.^6^ To quantify the nationwide burden of CL among HIV-positive and HIV-negative cases and the potential benefits of developing a new CL treatment, we constructed two stochastic Markov decision analytic computer simulation models in TreeAge Pro 2012 (TreeAge Software, Williamstown, MA): a CL Burden Model and a New CL Treatment Model. Both models focused on the societal perspective.

### Model structure: CL burden model.

One model, the Burden Model, focused on comparing the monetary burden of those with CL mono-infection to those with CL/HIV co-infection using the net present value (NPV), i.e., the sum of all the lifetime CL-related costs accrued by CL cases, adjusted to 2013 US$ costs using the discount rate. Throughout the remainder of our analysis, “CL/HIV co-infection” refers to infection by parasite species causing tegumentary leishmaniasis, which can present clinically as CL or MCL. Cost estimates generated by the model along with current national CL case reports^5^ and co-infection prevalence estimates^3^ were used to produce estimates of the total economic burden of CL in Brazil, including lifetime costs of currently infected cases.

Figure 1A

**Figure 1.**
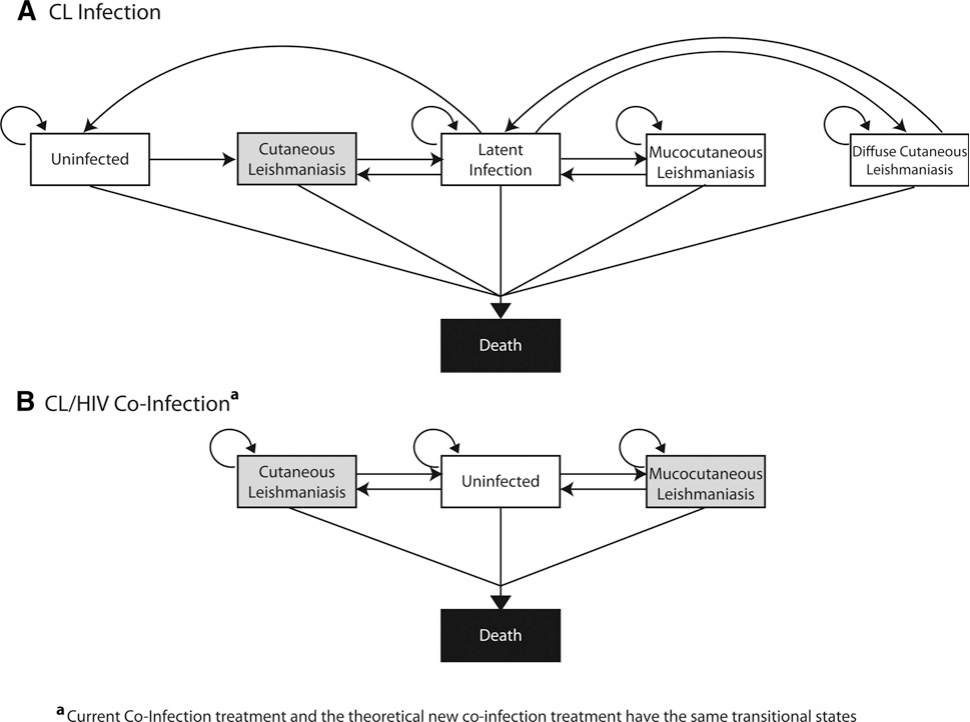
Model structure. (**A**) Markov health states and transition possibilities for human immunodeficiency virus (HIV)-negative individuals (cutaneous leishmaniasis [CL] Infection) as described in Bacon and others.^4^ (**B**) Markov health states and transition possibilities for HIV-positive individuals (CL/HIV Co-Infection). Gray shading indicates states where both CL and CL/HIV co-infection cases could begin.

and B illustrates the two branches of the CL Burden Model, CL Infection, and CL/HIV Co-infection, and the various ways individuals traveling through each branch could transition among Markov health states. Individuals with CL infection but without HIV could transition among six mutually exclusive Markov states:

- *Uninfected:* Healthy individuals not infected with CL, who have exceeded their risk for relapse.
- *Cutaneous Leishmaniasis* (*CL*)*:* Individuals were currently infected with CL and could only stay in this state for one year.
- *Latent Infection:* Individuals recovered from CL, MCL, or diffuse cutaneous leishmaniasis (DCL) and could stay in this state for a maximum of 10 years, until they died, or relapsed with CL, MCL, or DCL.
- *Mucocutaneous Leishmaniasis* (*MCL*)*:* Individuals were currently experiencing a more severe clinical presentation of *Leishmania* infection, affecting the oral and nasal mucosa, only able to occur after an initial CL infection.
- *Diffuse Cutaneous Leishmaniasis* (*DCL*)*:* Individuals were currently experiencing a more severe clinical presentation of *Leishmania* infection, where skin lesions were widely dispersed across the body, only able to occur after an initial CL infection.
- *Death:* Individuals died as a result of causes unrelated to *Leishmania* infection and were unable to continue cycling through the model.

A triangular distribution (mean: 37 years, range: 1–62 years) determined the age at which individuals entered the model infected with CL.^3^ During the first year, cases could be treated, cured, re-treated upon treatment failure, and cured after completion of the second treatment round. At the end of the first year, CL cases transitioned out of the CL state into either death or latent infection states. People could remain in the latter state for a maximum of 10 years and were at risk for MCL, DCL, or subsequent CL episodes. Individuals within the latent infection state were treated for CL, MCL, or DCL and are susceptible for a latent version of the infection. If no such infection occurs within 10 years, the individuals transition to the uninfected state and are considered cured. If MCL or DCL occurred, treatment with amphotericin B could result in side effects such as renal toxicity that had added costs attributable to extended hospital stay. Individuals then continued to cycle through the model until death as a result of unrelated causes. All probabilities and costs have been listed in Table 1.

By contrast, HIV-positive CL cases could transition among four mutually exclusive Markov states:

- *Uninfected:* HIV-positive individuals not infected with CL.
- *Cutaneous Leishmaniasis* (*CL*)*:* HIV-positive individuals infected with CL, disseminated throughout the body. Unlike HIV-negative CL cases, individuals were not required to leave this state after 1 year.
- *Mucocutaneous Leishmaniasis* (*MCL*)*:* HIV-positive individuals currently experiencing a more severe clinical presentation of *Leishmania* infection, affecting the oral and nasal mucosa. Unlike HIV-negative CL cases, these individuals did not need to have experienced a prior CL episode before developing MCL.
- *Death:* Individuals died as a result of causes unrelated to *Leishmania* infection and were unable to continue cycling through the model.

The HIV-positive individuals entered the model infected with either CL or MCL. Although in either of these states, individuals could transition to death, become uninfected, or remain in either CL or MCL states, shown in Figure 1B. Both CL and MCL health states had similar transition possibilities, but differing probabilities and costs associated with treatment, cure, and side effects (Table 1). As with HIV-negative cases, pentavalent antimonials were the first-line treatment of co-infected cases. Amphotericin and miltefosine were administered in the event of relapse or treatment failure. Once a case had experienced a treatment side effect, they could not receive the same medication again. Unlike HIV-negative individuals (CL infection branch), HIV-positive individuals (CL/HIV co-infection branch) had the probability of dying as a result of miltefosine treatment, although this probability was fairly small. The CL/HIV co-infection model has two fewer transitional states than the CL mono-infection because of the variations of the CL infection. The HIV-positive individuals contract a CL that disseminates throughout the body, with a different clinical presentation from both CL and DCL.^23^ Because there are no reported cases of HIV and DCL co-infection in Brazil, removal of DCL as a possible transitional state was necessary for those with co-infection.^3^,^23^ Additionally, the latent infection stage is not needed as it is extremely difficult to be cured of and the probabilities of contracting the infection at a subsequent time is not different between those who have not been infected to those who have previously been infected. If an individual is able to avoid relapse, they transition into the uninfected health state cured of CL and have the same probability of contracting CL as another HIV-positive individual. Co-infection has no latent infection state, as there is no mention of a latency period of infection for HIV and CL co-infected individuals in the literature.

### Model structure: new CL treatment model.

We developed a second model, the New CL Treatment Model, to determine the potential economic benefit of a new treatment of CL that would be more efficient at treating those co-infected with HIV (i.e., no hospitalization, no severe side effects). This model was also used to capture the economic impact of several uncertainties regarding the characteristics of the treatment and used results to establish potential thresholds and targets for drug development.

The New CL Treatment Model used the structure of the CL/HIV co-infection branch in the CL Burden Model (Figure 1B) to compare the current treatment described previously with a new CL treatment of those infected with HIV. Transition possibilities between health states were the same for both treatments; however, variations in treatments received resulted in differing costs and probabilities. The new treatment developed was assumed to not require hospitalization, thus hospital stay costs (i.e., cost of a hospital bed) were not included. Unlike the CL Burden Model, no other treatments were offered after the presentation of side effects, to ensure cost differences observed could be attributed to the new treatment and not existing second-line therapies.

### Model parameters.

A literature review was conducted using MEDLINE and the following terms: cutaneous leishmaniasis, human immunodeficiency virus, HIV, Brazil, mucocutaneous leishmaniasis, and co-infection. Baseline model costs and probabilities are listed in Table 1. Distributions from the literature were used to reflect the variability observed in reality. Single point values were used from the literature where little data was available. All costs were converted to 2013 US$ using a 3% discount rate.^24^ All treatment costs were based upon the World Health Organization (WHO) negotiated prices.^5^,^15^ The mortality rates included for the HIV-positive and negative populations were different, as the HIV-positive population had higher mortality rates for all ages.^3^,^25^

Initial treatment of HIV-negative CL cases included 20 mg/kg of pentavalent antimonials over a 20 day span, as recommended by WHO.^15^,^26^ Individuals were retreated with another regimen of pentavalent antimonials or pentamidine upon relapse^27^; after two treatment failures (i.e., two relapses), individuals no longer sought further treatment. The MCL or DCL cases initially received a 30-day regimen of pentavalent antimonials, although pentamidine and miltefosine were given if disease relapse occurred^13^; after four treatment relapses per MCL or DCL episode, treatment was discontinued.

The HIV-positive individuals who contracted CL required stronger medications and larger dosages. These cases initially received a total of 30 g of pentavalent antimonials administered throughout 30–60 days.^16^ If not cured or relapse occurred, 540 mg–1 g of amphotericin B was administered^16^; if treatment was unsuccessful or relapse occurred, patients were given 5,500 mg of miltefosine.^17^

Many drugs used to treat CL can be highly toxic and are associated with a variety of side effects that result in additional treatment costs. Renal toxicity is a side effect of Amphotericin B and was associated with a longer hospital stay (likeliest: 10 days, range: 1–127 days).^19^,^20^,^28^ Miltesfosine was also associated with side effects such as elevated serum urea nitrogen (BUN) and creatinine levels or elevated liver enzymes in addition to a small probability of death caused by treatment.^18^ As with CL infection, once an individual presented with a side effect, the medication was removed from their list of possible treatment options; however, there was no limit to the number of times HIV-positive individuals could seek treatment.

### Sensitivity analyses.

Sensitivity analyses were performed on several parameters in the CL/HIV co-infection branch of the CL Burden Model because of uncertainties regarding various treatment-related practices, such as the probability of seeking treatment (10–90%) among those co-infected with HIV. Additionally, few studies have quantified treatment efficacy or assessed the treatment-related costs associated with MCL, although lower cure rates for MCL than CL have been reported. Therefore, we ranged the ratio of the MCL cure rate to CL cure rate (from 10% to 90% of the CL cure rate) and the ratio of the MCL treatment cost to the CL treatment cost (from one to three times the cost of CL treatment cost). Current reports place case estimates at 26,008^5^ and HIV prevalence among CL cases around 0.1%^6^; however, it has been suggested that the true number of cases annually may be 2.8–4.6 times higher.^5^ To account for these uncertainties, the total number of CL cases and the CL/HIV co-infection prevalence were varied from 0.5 to 5 times current estimates.

For the New CL Treatment Model, MCL cure rate was assumed to be 70% of CL cure rate and MCL treatment was assumed to cost 1.5 times CL treatment cost. Sensitivity analyses performed on the new treatment branch included the probability of seeking treatment (10–90%), the cost of the new drug ($41–508, based on current co-infection treatment regimens), the probability of treatment side effects (1–30%), based on current treatment side effect probabilities), and the probability of treatment cure (30–100%).

## Results

### Overview.

Consideration of HIV co-infection boosted the NPV per CL case by 11–371 times to $1,349–45,683 compared with individuals with no co-existing infections ($123). These findings place the total economic burden of CL in Brazil over the lifetime of those currently infected at $3.23–4.38 million, assuming current CL/HIV co-infection rates and CL case estimates from Brazil. Assuming treatment-seeking behavior remains the same and treatment-seeking likelihood among those with co-infection is slightly higher than those with no co-infection (70%), a new CL drug therapy to address CL/HIV co-infection could be priced as high as $508 (two times the cost of pentavalent antimonials) and still save costs ($334 to $45,018 over the lifetime of each case). These results suggest that use of a new treatment could be cost saving at a price point of $254 or less across the range of treatments seeking probabilities, side effect risks, and cure rates evaluated and could continue to save costs up to a $508 price point across all treatment seeking probabilities as long as drug cure rate was ≥ 50%.

### CL burden model.

Table 2 shows results from the CL Burden Model, where ranges represent the variation observed across MCL cure rates. The columns represent different likelihoods of a CL case seeking treatment, a value that is determined by behaviors. Therefore, each column represents a different behavioral condition that could exist in the field and costs should only be compared within the same row. HIV co-infection increased the NPV of a CL case to $7,735, which is 63 times the NPV of a CL case without co-infection, assuming a 70% treatment-seeking likelihood and ratio of MCL treatment costs to CL treatment costs similar to HIV-negative cases (1.5) and a cure rate of 70% of that seen among cases without co-infection. Even at adoption rates lower than currently reported for CL infection (10%), a CL/HIV case could incur 11–29 times the cost of CL cases without HIV (additional cost of $1,226–3,463).

**Figure 2.**
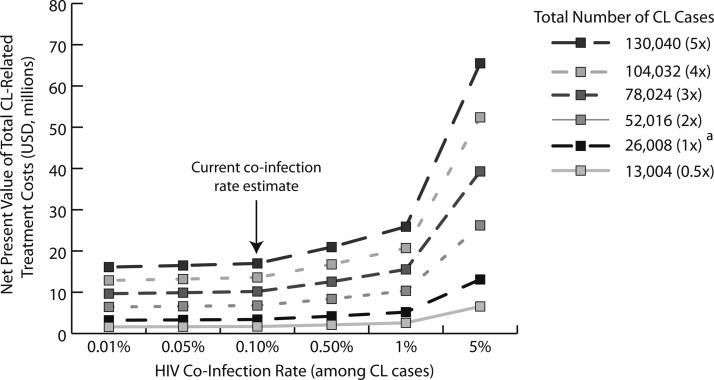
Net present value of total cutaneous leishmaniasis (CL)-related treatment costs in Brazil. * Number of CL cases reported annually from 2003 to 2007 in Brazil.^5^

Figure 2 shows the impact of CL/HIV co-infection prevalence and total CL case estimates on the economic burden of CL in Brazil over the lifetime of current cases, assuming MCL treatment costs are 1.5 times greater than CL costs, MCL cure rates are 70% of CL cure rates, and a treatment-seeking probability of 70%. Even if current estimates of CL cases in Brazil were cut by half to 13,004 and co-infection prevalence by 90–0.01%, the NPV of total CL treatment costs would still be over $1.6 million. Using current case CL and co-infection prevalence estimates yield an NPV of place this burden around $3.4 million, and underreporting rates suggested by some (2.8–4.6 times case reports) could increase the NPV to $15.6 million. Failing to consider these added costs associated with HIV co-infection when estimating the economic burden of CL in Brazil would yield an NPV of $1.6–16.0 million (assuming an NPV of $123 per case), which accounts for 99–24% of our nationwide estimates ($1.6–65.5 million) when considering HIV co-infection prevalence of 0.01–5% and a prevalence of 0.5–5 times the current reports.

### New treatment model.

**Figure 3.**
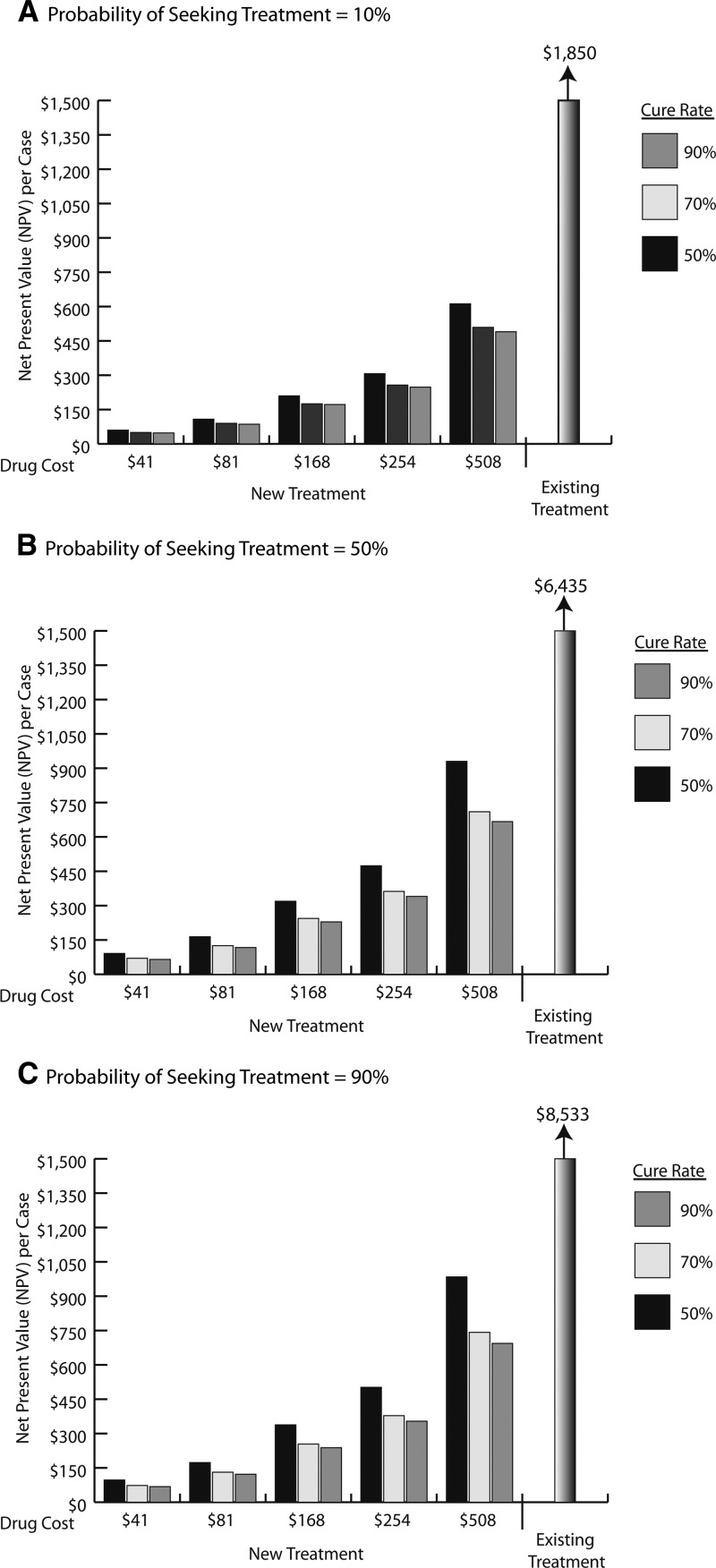
Net present value per case for the New Treatment compared with the Existing Treatment of various cure rates, drug costs, and probability of seeking treatment.

Figure 3 shows the lifetime costs per co-infection case using the new treatment (across the range of drug cost, treatment seeking likelihood, and cure rate) compared with the NPV of the existing treatment, assuming MCL treatment costs are 1.5 times greater than CL costs, MCL cure rates are 70% that of CL cure rates, and side effect likelihood of 10% (for only new treatment). These results suggest that use of a new treatment could be a cost saving ($1,238–8,464 lifetime costs per co-infection case) at a price point as high as $508 across all drug cure rates evaluated, regardless of the likelihood that treatment was sought. Side effect risk had a maximum impact when the probability of the case-seeking treatment was 90%, where increasing this probability from 1% to 30% increased the NPV per case by ∼2 times. Using the midpoint value assessed for drug cost ($168), side effect risk (10%), and cure rate (70%), the lifetime CL-related cost of an HIV-positive patient was $254, assuming a treatment-seeking probability of 70%. This cost is 30 times lower than the NPV per CL/HIV case ($7,735) using existing treatments and assuming the same treatment seeking likelihood, resulting in a total savings of $7,481 per case.

**Figure 4.**
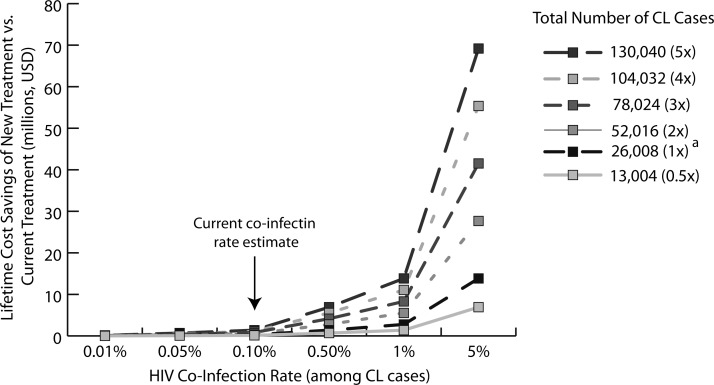
Cost savings of development and use of new treatment instead of current treatment of cutaneous leishmaniasis/human immunodeficiency virus (CL/HIV) co-infection over the lifetime of current cases in Brazil. Number of CL cases reported annually from 2003 to 2007 in Brazil.^5^

Figure 4 illustrates the potential cost savings associated with use of a new drug therapy developed for CL/HIV co-infection (instead of the currently available treatment) over the lifetime of those currently infected with CL in Brazil. According to current case reports, the new treatment could save between $0.02 and $9.7 million depending on CL/HIV co-infection rate, but could save $0.10–48.6 million (∼5 times current estimates) assuming 130,040 cases of CL depending on co-infection rate.

For the New CL Treatment Model, MCL cure rate was assumed to be 70% of the CL cure rate and MCL treatment was assumed to cost 1.5 times CL treatment cost. Sensitivity analyses performed on the new treatment branch included the probability of seeking treatment (10–90%), the cost of the new drug ($41–508, based on current co-infection treatment regimens), the probability of treatment side effects (1–30%), based on current treatment side effect probabilities), and the probability of treatment cure (30–100%). Ranging the drug cost (i.e., price) helped capture the possibility that research and developmental costs would be rolled into the drug price to varying degrees.

## Discussion

Our study quantifies the substantial degree to which HIV co-infection increases the burden of CL and shows the importance of other associated infections, especially those that affect the immune system, when considering the impact and control of an infectious disease. Some efforts have emerged to account for the interplay between HIV and CL such as the 2002 emergence of the Brazilian *Leishmania*-HIV Co-infection Network^6^ with goals of mapping the geographical distribution of *Leishmania*-HIV co-infection to gain further understanding of the clinical presentation and features, and identify the most effective methods of diagnosis. However, in general, reported co-infection numbers tend to be underestimates because leishmaniasis is not currently among the Centers for Disease Control and Prevention's list of HIV/acquired immunodeficiency syndrome (AIDS) opportunistic infections and therefore are often not reported to formal AIDS surveillance networks.^6^

Our study may in fact underestimate the impact of the interplay between HIV and CL. It focused solely on the effects of HIV on CL and did not consider how CL may increase HIV costs. For example, 42–68% of HIV-positive also experienced another form of opportunistic infection during their VL episode,^29^,^30^ likely increasing medical costs and mortality risk. *Leishmania* parasite amastigote surface molecules additionally enhance HIV transcription rate, further suppressing the immune system, lowering CD4 counts, and causing them to progress more rapidly to acquired immunodeficiency syndrome (AIDS).^31^ Moreover, our study did not consider that HIV immunosuppression increases susceptibility to *Leishmania* infection, thereby increasing the prevalence of *Leishmania* infection, and that the presence of CL can subsequently worsen HIV infection.

Currently, available drugs recommended for CL treatment are associated with several drawbacks and limitations, such as lengthy regimens, and potentially irreversible damage to the pancreas, kidneys, or bone marrow.^32^ Outcomes of use of existing therapies among HIV-positive patients are even more severe, as treatments among those who are immunocompromised are often associated with higher rates of kidney and liver dysfunction and occasionally death.^18^ Additionally, infections with the cutaneous form of disease have been reported to visceralize and spread to the vital organs in HIV-positive cases^33^ and several drugs administered to HIV-positive persons often increase *Leishmania* treatment toxicity, which are both complications not captured by our study.^2^ Given these difficulties and setbacks, the development of a new drug or new formulation of an old drug, which is more effective at treating immunosuppressed CL cases is necessary. Although our study did not consider productivity losses as a result of lost wages during treatment, treatment duration was responsible for a large portion of overall treatment burden, as the cost of hospital stay made up 70–80% of total costs across existing treatment options in our model. As shown by our results, a drug therapy not requiring hospital stay (as was assumed for our new treatment) could be highly cost saving, even at higher drug prices. Our economic analysis of CL treatment among the HIV-positive population in Brazil can be informative for drug developers, funders, government officials, and decision makers in identifying a TPP for new drug therapies.

There is a relative dearth of new treatment CL options being developed, which may be caused by the lack of attention and resources currently allotted to controlling and treating CL. Our study findings suggest that more attention toward developing new CL treatments may be warranted. The development of a new treatment could be extremely beneficial to Brazil. In contrast to the estimated $3.4 million accrued by current CL cases nationwide (assuming current CL case and co-infection estimates), Brazil had a health expenditure of over $710 billion between 2008 and 2012.^34^ Additionally, in 1995, Brazil spent $205 million on the treatment of Chagas' disease, an infection that can cause long-term damage to the heart and other organs.^35^ By developing a new and effective treatment of those with co-infection, several million dollars could be saved over the remaining lifetime of those currently infected.

## Limitations

Models, by definition are simplifications of a decision, process, or system, designed to help better understand the situation and its key relationships, and cannot possibly capture every single factor or possibility.^36^,^37^ Limited information regarding CL/HIV co-infection required various assumptions to be made. For instance, studies have reported MCL in the HIV-negative population as being 1.5 times more expensive than CL^11^,^38^; however, this may differ in co-infected individuals. As comprehensive breakdown of CL treatment-associated costs in Brazil was not found, our model remained conservative, only including drug costs, cost of hospital stay, and a small materials cost. Additionally, because the drug miltefosine is not marketed in Brazil, its price cannot be easily calculated but has been given a range of costs based upon previously published literature.

Our study used estimates of CL prevalence among the HIV-positive population in Brazil and varied this value to account for uncertainty and does not make assumptions regarding CL infection risk among the HIV-positive population as a whole. Although CL/HIV co-infection has been reported in several countries around the world, our analysis focused on Brazil, because of the amount of country-specific data in the literature. However, these models could be used to conduct similar analyses elsewhere as more data becomes available.

Although it has been noted that the lower the CD4 count in an individual, the worse the CL infection, all HIV-positive individuals with CL were treated similarly in our models.^6^ Individuals with both HIV and CL are considered to be in stage 4 of HIV, typically indicating a low CD4 count, grouping these individuals together while being a slight oversimplification, should be an accurate representation of the group as a whole.^6^

## Conclusion

Neglecting the interplay between HIV and CL infections may lead to an underestimate of their respective impact. The HIV co-infection boosted the NPV per CL case by 11–371 times to $1,349–45,683 compared with individuals with no co-existing infections ($123) and thus the national burden of CL in Brazil from $1.6–16.0 million to $1.6–65.5 million depending on the co-infection rate. Results indicate that the use of a new CL treatment not requiring hospitalization could reduce the economic CL burden in Brazil by $0.20 million at the current reported number of CL cases and percent co-infection rates and can save as much as $1.0 million and $9.7 million at higher CL incidence rates and co-infection rates, respectively, across a range of drug profiles. In addition to the economic benefits for the development of more effective CL therapies for both co-infection and mono-infection, these findings suggest the need for joint HIV and CL surveillance and control efforts as well.

## Figures and Tables

**Table 1:** Model inputs

Parameter		Value	Reference
CL infection			
Lifetime risk	MCL	2%	9,10
	DCL	6%	11
Cure rate	Pentavalent antimonials (CL)	61%* (40–86%)	12
	Pentavalent antimonials (MCL)	67%* (28–94%)	13
	Pentamidine (CL)	75%* (71–87%)	13
	Pentamidine (MCL)	93%	13
	Amphotericin B (MCL)	89%	13
	Any treatment (DCL)	(0–10%)†	14
Cost‡	Pentavalent antimonials (CL)	$169.37	15
	Pentavalent antimonials (MCL/DCL)	$254.06	15
	Pentamidine (CL/MCL/DCL)	$0	15
	Amphotericin B (MCL/DCL)	$150	15
Seeking treatment	CL cases	20–60%†	4
	MCL/DCL Cases	40–100%†	4
CL/HIV co-infection			
Probabilities	CL and HIV	32%	3
	MCL and HIV	68%	3
Cure rate	Pentavalent antimonials (CL)	50–100%†	16
	Amphotericin B (CL)	90%* (75–100%)	16
	Miltefosine (CL)	64%* (50–100%)	17
Relapse rate	Pentavalent antimonials (CL)	75–100%†	16
	Amphotericin B (CL)	25%* (0–100%)	16
	Miltefosine (CL)	88%* (75–100%)	17
Side effects	Elevated liver enzymes (Miltefosine)	1–10%†	18
	Elevated BUN and creatinine (Miltefosine)¶	0.01–1%	18
	Death (Miltefosine)	0.9%	18
Cost	Pentavalent antimonials (CL)	$254.05	15,16
	Amphotericin B (CL)	$81–150†	15,16
	Miltefosine (CL)	$119–144†	17
Used for CL infection and CL/HIV co-infection			
Side effects	Renal toxicity (Amphotericin)	15%	19,20
Cost	Laboratory materials (per day)	$0.50	21
	Hospital bed (per day)	$18.67	22

* Triangular distribution.

† Uniform distribution.

‡ All costs are in 2013 US dollars (US$).

¶ Both serum urea nitrogen (BUN) and Creatinine levels are used to determine kidney functionality.

MCL = mucocutaneous leishmaniasis; DCL = diffuse cutaneous leishmaniasis; CL = cutaneous leishmaniasis; HIV = human immunodeficiency virus.

**Table 2:** Lifetime cost per CL/HIV co-infection case*

Ratio of MCL treatment costs to CL treatment costs	Probability of seeking treatment				
	10%	30%	50%	70%	90%
1.0	$1,349–1,484	$3,096–4,400	$4,130–7,760	$4,751–11,754	$5,146–16,434
1.5	$1,805–2,010	$4,217–6,142	$5,669–11,033	$6,539–16,863	$7,134–23,763
2.0	$2,263–2,533	$5,350–7,893	$7,221–14,313	$8,332–21,997	$9,103–31,050
2.5	$2,725–3,056	$6,471–9,638	$8,763–17,571	$10,148–27,153	$11,042–38,372
3.0	$3,179–3,586	$7,581–11,401	$10,329–20,848	$11,924–32,208	$13,001–45,683

* CL = cutaneous leishmaniasis; HIV = human immunodeficiency virus; MCL = mucocutaneous leishmaniasis.
